# Reproductive care in Thai women with diabetes mellitus: a descriptive cross-sectional study

**DOI:** 10.1186/s12978-023-01694-w

**Published:** 2023-10-12

**Authors:** Kritat Pothongsangarun, Jiayu Li, Witthawat Naeowong, Chayanis Apirakviriya, Phanupong Phutrakool, Tunchanok Juntamongkol, Thita Sae-chueng, Kantasorn Horpratraporn, Unnop Jaisamrarn, Somsook Santibenchakul

**Affiliations:** 1 Department of Obstetrics and Gynecology, Faculty of Medicine, Chulalongkorn University, Bangkok, Thailand; 2 Department of Internal Medicine, Faculty of Medicine, Chulalongkorn University, Bangkok, Thailand; 3 Department of Obstetrics and Gynecology, King Chulalongkorn Memorial Hospital, Bangkok, Thailand; 4 Chula Data Management Center, Faculty of Medicine, Chulalongkorn University, Bangkok, Thailand; 5 Faculty of Medicine, Chulalongkorn University, Bangkok, Thailand; 6 Center of Excellence in Menopause and Aging Women Health, Department of Obstetrics and Gynecology, Faculty of Medicine, Chulalongkorn University, Bangkok, Thailand; 7 Center of Excellence in Preventive & Integrative Medicine, Faculty of Medicine, Chulalongkorn University, Bangkok, Thailand

**Keywords:** Preconception care, Sexual and reproductive planning, Contraception, Diabetes

## Abstract

**Background:**

Pre-existing diabetes mellitus (DM) is a challenging pregnancy complication as poor glycemic control is associated with adverse maternal and fetal outcomes. In this study, we aimed to investigate DM-related knowledge, attitudes, preconception care practices, and contraceptive prevalence in women with DM.

**Methods:**

This descriptive cross-sectional survey was conducted among reproductive-aged Thai women receiving DM treatment at King Chulalongkorn Memorial Hospital between August 1, 2021, and June 30, 2022. Patients with DM who were not pregnant or trying to conceive and could be contacted via the phone were included and a validated self-administered questionnaire was distributed electronically.

**Results:**

A total of 238 participants were included in the final analysis, yielding 69.4% response rates. The mean (standard deviation) score for knowledge of pregnancy planning and pregnancy-related risks was 6.8 (3.5) out of 15. Only about half of the participants had discussed pregnancy planning with their physicians. Multivariable analysis showed that younger age at DM diagnosis, non-Buddhism, married, higher education, and medical personnel were significantly associated with higher knowledge scores. Women aged > 45 years and those with higher practice scores had significantly higher adjusted odds of using highly effective contraception; the most common methods included male condoms and combined oral contraceptive pills. There was an unmet need for contraception in 9.5% of women with DM.

**Conclusions:**

Although highly effective contraception is safe for patients with DM, only about half of our participants used tier one or two contraceptives or had received consultation regarding preconception planning. There was a notable gap in care coordination among specialists; integrating reproductive healthcare into DM therapy would improve access to preconception care.

**Supplementary Information:**

The online version contains supplementary material available at 10.1186/s12978-023-01694-w.

## Plain language summary

We aimed to study the knowledge, attitudes, preconception care practices, and contraceptive usage among women with diabetes mellitus (DM) who were of reproductive age. A survey was conducted among Thai women receiving DM treatment at a university hospital. The results showed that participants had limited knowledge about pregnancy planning and diabetic-related risks. Only half of them had discussed pregnancy planning with their doctors. Factors such as younger age at DM diagnosis, being non-Buddhism, being married, having higher education, and being medical personnel were associated with higher knowledge scores. Older women and those with better practice scores were more likely to use highly effective contraception. However, there was still a need for contraception in some women with DM. We concluded that there was a lack of integration between specialists in reproductive healthcare and DM therapy, and improving this coordination would enhance access to preconception care for women with DM.

**Supplementary Information:**

The online version contains supplementary material available at 10.1186/s12978-023-01694-w.

## Introduction

Diabetes mellitus (DM), a hyperglycemic state resulting from improper glucose metabolism, is a chronic disease that significantly influences the lives and well-being of individuals worldwide [1]. The International Diabetes Federation projects that the global prevalence of DM will increase from 10.5% (536.6 million people) in 2021 to 12.2% (783.2 million people) in 2045 [1]. In 2021, it was estimated that 10.2% of women worldwide aged 20–79 have DM [1]. According to the Thai National Health Examination Survey 2004–2014, the prevalence of DM in Thai adults older than 20 years is 9.9%, with a higher prevalence among women [2]. The prevalence of DM is increasing in Thai women. Furthermore, women in Thailand have a higher proportion of undiagnosed DM than men, reaching up to 45% of women [2].

Pre-existing DM primarily refers to type 1 or 2 DM diagnosed before pregnancy, which complicates 1–2% of all pregnancies and accounts for 13–21% of DM cases in pregnancy [3, 4]. This condition substantially threatens maternal and perinatal outcomes, including miscarriage, congenital anomalies, preterm birth, preeclampsia, and increased perinatal morbidity and mortality [5–7]. An increase in hemoglobin A1c (HbA1c) levels from 5.6 to 6.8% can triple the risk of congenital abnormalities [8, 9]. Good glycemic control before conception reduces the incidence of congenital anomalies and may mitigate the risk of preterm birth [10]. Preconception care improves glycemic control in early pregnancy, thus minimizing poor pregnancy outcomes [10]. The American Diabetes Association recommends that routine diabetic care by maintaining HbA1c levels at lower than 6.5% is essential to reduce adverse pregnancy outcomes among all diabetic women with reproductive capability before conception and throughout pregnancy [11]. Another crucial component of preconception care is ensuring that women use effective contraception until their treatment regimen and HbA1c are optimized for pregnancy [11].

Medical societies recommend at least annual preconception care for all women of reproductive age who have DM since they may be at risk of pregnancy between visits [12]. All women of reproductive age with type 1 or 2 DM should be educated about the potential consequences of DM, the effect of its medications on maternal and fetal outcomes, and the impact of pregnancy on their DM treatment and co-existing conditions. Standard DM care should include contraceptive counseling, which is fundamental to sexual and reproductive health care. Despite numerous efficient contraceptive options, women with DM tend to use no contraception compared to those with normoglycemia [13]. In addition, they tend to receive less guidance on long-acting reversible contraception (LARC) initiation, despite medical societies recommending the safety of LARC use among women with DM [7, 8, 14]. Contraceptive implants and intrauterine devices (IUDs), classified as LARC, have particular significance for women diagnosed with diabetes mellitus due to their established safety profile, capacity to mitigate user-related errors, and ability to offer more reliable contraception [15, 16]. By minimizing the likelihood of unwanted pregnancies that may lead to difficulties, these methods can effectively contribute to the overall well-being of women with diabetes mellitus. Although there is an increasing trend of DM among Thai women, few studies have investigated preconception care and contraceptive prevalence in this population [17]. Accordingly, we aimed to explore the knowledge, attitudes, and practices regarding perception planning among Thai women of reproductive age. We also investigated the prevalence and factors associated with contraceptive use.

## Methods

### Study setting and design

We conducted a descriptive cross-sectional study at the King Chulalongkorn Memorial Hospital (KCMH) from August 1, 2021, to June 30, 2022. It is a tertiary referral hospital in Bangkok, Thailand, and a residency training site for many clinical areas. KCMH serves approximately 1200–1500 diabetic outpatients each year. Patients with non-complicated type 2 DM attended the Internal Medicine Outpatient Clinic. In contrast, all patients with type 1 DM, complicated type 2 DM, and some other DM patients attended the Internal Medicine and Diabetic, Thyroid, and Endocrinology Outpatient Clinic. The KCMH preconception and family planning clinics are only available by appointment rather than on a walk-in basis. The clinic offers the following contraceptive methods: combined oral contraceptive pills (COCs), progestin-only pills (POPs), depot medroxyprogesterone acetate (DMPA), levonorgestrel and etonogestrel contraceptive implants, copper intrauterine device (copper IUD), levonorgestrel intrauterine system (LNG-IUS), male and female sterilization, and condoms. LARC is free of charge for adolescents and includes levonorgestrel or etonogestrel implants and copper IUD. Moreover, those who physically resided in Bangkok were partially reimbursed for LARC in accordance with the Thailand National Health Security policy. Healthcare programs cover female sterilization to varying degrees. Other forms of contraception were not covered by health insurance programs. In Thailand, combined hormonal contraceptives and POPs are available in pharmacies without a prescription.

### Study participants and eligibility

Thai women aged 18–49 years with a minimum one-year history of DM were eligible for this study. We included all reproductive-aged women regardless of their current sexual activity status to align with guidelines advocating preconception care for all women with diabetes mellitus.

Patients with type 1 and 2 DM (I10.x according to the International Classification of Diseases-10) were enrolled [18]. Women were excluded if they met any of the following conditions: (1) an unspecified type of diabetes, (2) could not be reached by the registered phone contact, (3) being pregnant or trying to conceive, (4) a history of hysterectomy and/or bilateral salpingo-oophorectomy, (5) had reached natural menopause, or (6) were not interested in participating in the survey. Each participant was compensated with 300 THB (approximately 10 USD) for their time after completing the questionnaire.

### Sample size

The sample size was determined using the finite population proportion formula and the following parameters: proportion of contraceptive usage among Asians with diabetes, 82.1% [13]; confidence interval, 95%; and margin of error, 5%. The total sample size was calculated to be 226. We chose to focus on contraceptive prevalence as it provides a real-world indicator of the effectiveness of preconception care delivery in this patient population. This approach allows us to evaluate how well preconception care practices are being implemented and received.

### Measurement tools

Based on an extensive literature review, we developed a self-administered questionnaire in the Thai language [13, 19–22] and assessed its content validity and reliability. This questionnaire was validated by an endocrinologist (WN) and two family planning experts (SS and UJ). All items attained an item-level Content Validation Index (I-CVI), and the scale-level Content Validation Index (CVI) reached scores exceeding 0.8. Additionally, a pilot comprehension test involving 10 volunteers was conducted to enhance the clarity of the survey questions. The test–retest reliability analysis revealed reliabilities of 0.8 and 0.9. The demographics, reproductive history, diabetic status and other comorbidities, knowledge, attitudes, and practices regarding preconception care were covered in this five-part questionnaire. Age, address, religion, reimbursement, marital status, highest educational attainment, occupation, income, number of living children, and contraceptive history were all included in the demographic and reproductive history information [19]. Age at DM diagnosis, disease activity, DM-related complications, current DM medication, and other comorbidities were all considered when determining DM status and other comorbidities [19]. This section’s information was confirmed with the participant's electronic medical record (EMR), and the discrepancy was verified based primarily on the data from the EMR. Knowledge regarding preconception planning was divided into two parts. The first section, primarily based on knowledge of pregnancy planning and pregnancy-related risks, included 13 true/false questions and two fill-in-the-blank questions about the optimal level of glycemic control before conception [21]. We scored one point for each correct answer and zero for each incorrect answer or response to “Do not know.” The second section included ten true/false questions regarding the safe use of each contraceptive in patients with DM [9]. The attitude section included four subtopics: susceptibility to negative outcomes of sexual activity, severity to negative maternal and perinatal outcomes of sexual activity, benefits of preconception planning, and barriers to preconception care and access to contraceptives, with responses ranging from ‘least likely’ (scored 1) to ‘most likely’ (scored 5) [20]^.^ Concerning the first two components, a higher attitude score indicated greater concern for individual health. A higher attitude score in the third component represented awareness about the benefits of preconception care. In the final section, the higher the attitude score, the greater the participant’s concern about access to preconception care and contraceptives. The practice section consisted of eight questions about the participant’s experience with sexual and reproductive planning, with the same scoring system as the knowledge and attitude section for the first five questions [13, 20, 22]. The other three questions used multiple-choice answers to inquire about participants’ experiences.

### Data collection and management

**Fig. 1:**
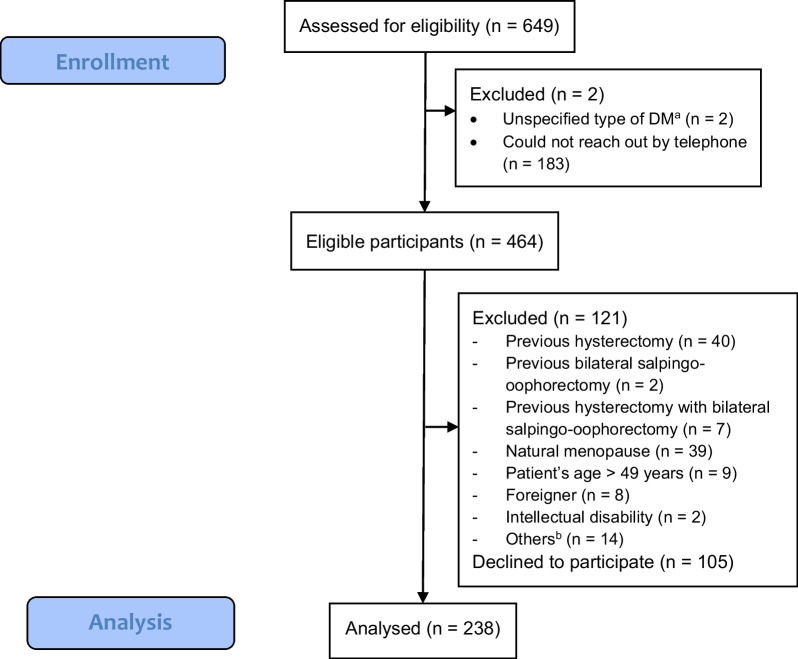
Participants recruitment flowchart. a Categorized from electronic medical record according to International Classification of Diseases-10 (ICD-10) code. b Others including blindness, pituitary tumor with post-surgery, intellectual disability, death, and wrong code of diagnosis

From April 1, 2021, to April 30, 2022, a list of reproductive-aged diabetic female patients at KCMH’s Internal Medicine and Diabetic, Thyroid, and Endocrinology Outpatient Clinic was retrieved and compiled by verifying outpatient records using the International Classification of Diseases, Tenth Revision. In total, 649 women with DM were included in the eligibility evaluation list. A brief telephone interview was conducted to determine eligibility. If the potential participants did not respond to the initial call, five attempts were made to contact them. Finally, our study included 238 eligible DM patients (Fig. 1). Three research assistants gathered the DM-related clinical data from the KCMH’s EMRs. Participants were asked to complete a self-administered questionnaire using the smartphone application “Line official account [23]”. Those unfamiliar with the electronic questionnaire were given an appointment for a self-administered questionnaire at the KCMH family planning clinic. Before beginning the study, all research team members were trained in participant recruitment, obtaining informed consent, and data collection. The principal investigator (KP) checked the correctness of the data abstraction according to the research protocol. We managed online surveys and databases using Research Electronic Data Capture (REDCap) Software, hosted at Chulalongkorn University’s Faculty of Medicine [24, 25].

### Variables

Contraceptive use was defined as any contraceptive method used over the past 12 months. The contraceptive method was classified by its effectiveness: highly effective contraception was defined as contraceptive methods that cause less than one pregnancy per 100 women in a year (male and female sterilization, contraceptive implant, and IUD); moderately effective contraception was defined as contraceptive methods that caused one to 12 pregnancies per 100 women per year (COCs and patch, POPs, and DMPA); less effective contraception was defined as contraceptive methods leading to less than 18 pregnancies per 100 women in a year (male condoms, female condoms, withdrawal, and fertility awareness [26]. The modern contraceptive method is identified as a product or medical procedure that interferes with reproduction after sexual intercourse [26]. If a participant used dual methods, the most effective method was considered the primary contraceptive. According to the American College of Obstetricians and Gynecologists, tier one and two contraceptives include sterilization, hormonal contraception, and lactation amenorrhea [27]. An unmet need for contraception referred to fertile women who were not taking any contraception but desired to delay the subsequent pregnancy [28]. Contraceptive prevalence was specifically calculated for those sexually active, defined as engaging in sexual activities with a male partner in the past 12 months, consistent with the study’s 12-month time frame for assessing contraceptive use.

### Data analysis

Data analysis was performed using STATA version 17 (StataCorp. 2021. Stata Statistical Software: Release 17. College Station, TX: StataCorp LLC.). Means with standard deviation (SD) and medians with interquartile range (IQR) were reported for normally and non-normally distributed data, respectively. Frequency and percentage were used to summarize categorical data.

A linear regression analysis was performed to explore the associations of demographics and clinical factors with the knowledge scores, focusing on pregnancy planning and pregnancy-related risks and reporting the mean difference and 95% CIs. We conducted a logistic regression analysis to investigate the factors associated with highly effective contraceptive use only among participants who reported sexual activity within the past 12 months and with the sub-questions pertaining to contraceptive practice; the results are presented as odds ratios (ORs) with 95% CIs. All significant variables with p-values less than 0.20 in the univariable regression analysis were included in the multivariable model. A two-way p-value of less than 0.05 was considered statistically significant.

## Results

A total of 238 participants were included in the final analysis, yielding 69.4% (238/349) response rates, as shown in Fig. 1. Among them, 127 participants were from the Outpatient Department of Internal Medicine, and 111 were from the Diabetic, Thyroid, and Endocrinology Outpatient Clinic. Six participants, who were unfamiliar with the electronic questionnaire, completed a self-administered questionnaire at the KCMH Family Planning Clinic.

### Sociodemographic and clinical characteristics

Basic participant characteristics are presented in Table 1. The mean (SD) age among participants was 39.5 (7.9) years. The majority of respondents in our study resided in Bangkok (56.3%), were Buddhist (95.8%), held a bachelor’s degree or higher (65.1%), and had never had children (53.4%). Most women (26.1%) were covered by the Social Health Insurance Scheme and the Universal Health Coverage Scheme (24%). Most women (55.9%) stated that they were married or cohabiting. The median (IQR) age at diagnosis of DM among all participants was 32 (20) years. The most common DM-related complications were diabetic ophthalmopathy (16%) and diabetic nephropathy (15.6%). The most common associated comorbidities were hypertension (39.9%) and dyslipidemia (39.1%) [See Additional file 1: Table S1].

**Table 1:** Socio-demographic and obstetric characteristics of women with diabetes (N = 238)

Variables	Total (N = 238) n (%)
Age	
Mean ± SD	39.5 ± 7.9
Median (IQR)	41.5 (12)
Age group	
≤ 19 years	1 (0.4)
> 19–24 years	13 (5.4)
> 24–35 years	56 (23.5)
> 35–45 years	103 (43.3)
> 45 years	65 (27.3)
Address	
Bangkok	134 (56.3)
Others^a^	104 (43.7)
Religions	
Buddhism	228 (95.8)
Christianity	3 (1.3)
Islam	6 (2.5)
No religion	1 (0.4)
Reimbursement	
Universal Coverage Scheme	57 (24)
Social Health Insurance Scheme	62 (26.1)
Self-reimbursement	23 (9.7)
King Chulalongkorn Memorial Hospital Officers	49 (20.6)
Civil Servant Medical Benefit Scheme	40 (16.8)
Missing data	7 (2.9)
Marital status	
Married or cohabiting	133 (55.9)
Single	93 (39.1)
Divorced/Widow	12 (5)
Highest education attainment	
Primary	6 (2.5)
Secondary	42 (17.7)
Vocational	35 (14.7)
Bachelor's degree or higher	155 (65.1)
Occupation	
Professional/ skilled^b^	129 (54.2)
Unskilled^c^	46 (19.3)
Unemployed^d^	63 (26.5)
Number of Living children	
0	127 (53.4)
1	56 (23.5)
2	49 (20.6)
3	6 (2.5)

^a^Including other provinces in Thailand

^b^Including healthcare professionals, office workers, and civil servant

^c^Including self-employed

^d^Including students and others

### Knowledge, attitude, and practice regarding preconception care

**Fig. 2:**
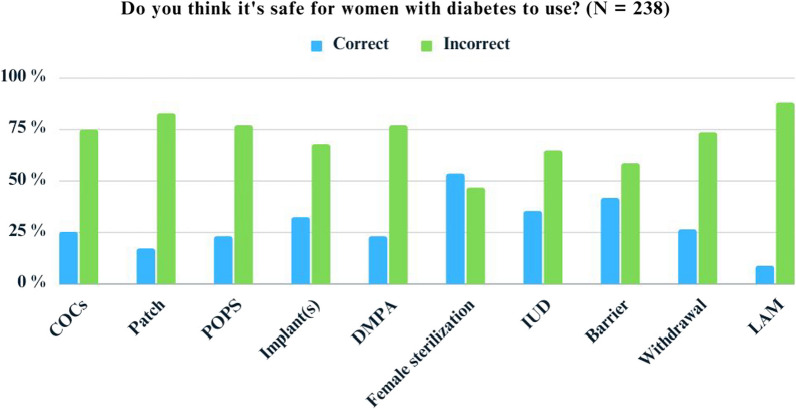
Proportion of respondents who correctly answered the question on the safe use of each contraceptive. COCs = Combined oral contraceptive pills. POPs = Progesterone-only pills. DMPA = Depot medroxyprogesterone acetate. IUD = intrauterine device. LAM = Lactation amenorrhea

The mean (SD) score of preconception care score focusing on pregnancy planning and pregnancy-related risks was 6.8 (3.5) out of 15. The percentage of correct responses to the knowledge questions regarding pregnancy planning and pregnancy-related risks is shown in Additional file 1: Table S2. Less than half of our participants knew that insulin was safe during pregnancy. Figure 2 depicts the percentage of correct responses regarding the safety of each contraceptive among patients with DM. The methods with the most correct responses were tier one and two; however, only approximately half responded correctly. Table 2 shows the factors associated with knowledge scores focusing on pregnancy planning and pregnancy-related risks by univariable and multivariable linear regression analyses. Multivariable analysis showed that increasing age at the time of diagnosis was significantly associated with lower knowledge scores. Compared to those whose DM diagnosis was made at an age equal to or less than 35 years, those whose DM diagnosis was made at the age > 35–45 years and > 45 years had significantly lower knowledge scores of which the adjusted mean difference and (95% CIs) were − 1.17 (− 2.05, − 0.29) and − 2.01 (− 3.80, − 0.38), respectively. Religion, marital status, education, and occupation were significantly associated with knowledge scores in multivariable analysis. Buddhist participants had lower knowledge scores compared to other groups, with an adjusted mean difference (95% CI) of − 2.44 (− 4.41, − 0.46). Compared to single participants, married or cohabiting participants had significantly higher knowledge scores, with an adjusted mean difference (95% CI) of 1.91 (1.09, 2.73). The adjusted mean difference and 95% CI of participants who earned a bachelor's degree or higher was 1.78 (0.93, 2.63) compared to those who earned a lower degree. Compared to non-medical personnel, those who were medical personnel had significantly higher knowledge scores, of which the adjusted mean difference and (95% CI) were 1.35 (0.25, 2.45).

**Table 2:** Factors influencing pregnancy planning and pregnancy-related risk knowledge scores by linear regression (N = 238)

Factor	Univariable model		Multivariable model^a^	
	Mean difference (95% CI)	P-value	Mean difference (95% CI)	P-value
Type of DM				
Type 1	− 0.21 (− 1.62, 1.21)	0.78		
Type 2	Reference			
Age group				
≤ 35 years	Reference			
> 35–45 years	0.26 (− 0.78, 1.30)	0.62		
> 45 years	− 0.32 (− 1.47, 0.84)	0.59		
Age at diabetes diagnosis				
≤ 35 years	Reference		Reference	
> 35–45 years	− 0.92 (− 1.85, 0.01)	0.05	− 1.17 (− 2.05, − 0.29)	< 0.01
> 45 years	− 2.11 (− 3.95, − 0.26)	0.03	− 2.09 (− 3.80, − 0.38)	0.02
Address				
Bangkok	0.50 (− 0.38, 1.37)	0.26		
Others^b^	Reference			
Religions				
Buddhism	− 2.12 (− 4.26, 0.02)	0.05	− 2.44 (− 4.41, − 0.46)	0.02
Others^c^	Reference		Reference	
Reimbursement				
Universal Coverage Scheme	− 2.09 (− 3.57, − 0.61)	0.01		
Social Health Insurance Scheme	− 0.56 (− 2.01, 0.90)	0.46		
Self-reimbursement and missing data	Reference			
King Chulalongkorn Memorial Hospital Officers and Civil Servant Scheme	− 0.33 (− 1.71, 1.05)	0.64		
Marital status				
Married	1.41 (0.55, 2.26)	< 0.01	1.91 (1.09, 2.73)	< 0.01
Single or divorced	Reference		Reference	
Highest education attainment				
Lower than tertiary level	Reference		Reference	
Bachelor’s degree or higher	1.57 (0.68, 2.46)	< 0.01	1.78 (0.93, 2.63)	< 0.01
Occupation and current employment status				
Others^d^	Reference		Reference	
Professional/ skilled^e^	1.29 (0.12, 2.46)	0.03	1.35 (0.25, 2.45)	< 0.01
Current contraceptive method				
Yes	1.20 (0.31, 2.10)	0.01		
No	Reference			
Comorbidities^f^				
Yes	0.71 (-0.44, 1.85)	0.22		
No	Reference			
No data	− 1.39 (− 2.34, − 0.44)	< 0.01		

^a^Adjusted multivariable model including age at diabetes diagnosis, religions, marital status, education, and occupation and current employment status

^b^Including other provinces in Thailand

^c^Including Christianity, Islam, and no religion

^d^Including students, self-employed, office workers, civil servants, and others

^e^Including healthcare professional

^f^Including diabetic ophthalmopathy, diabetic nephropathy, diabetic neuropathy, ischemic and hemorrhagic stroke, and peripheral arterial disease

Additional file 1: Table S3 displays the attitudes of women with DM regarding preconception care. Participants were concerned about their health and had immense awareness of the benefits of preconception care. They had some concerns about access to preconception care and contraceptives. Additional file 1: Table S4 shows the participants’ experiences regarding sexual and reproductive planning. The median (IQR) score of practice towards confidence in family planning practice was 2 (2). About half of the participants (53.8%) had discussed pregnancy planning with their internists or endocrinologists. Half of the individuals (51.7%) chose contraception based on recommendations from healthcare professionals. We conducted a subgroup analysis of participants who reported sexual activity in the previous 12 months and found consistent results.

### Contraceptive prevalence

About 70% of participants (168/238) were sexually active in the past 12 months. The contraceptive prevalence among this group was 90.5%, as shown in Table 3. The modern contraceptive prevalence was 81.6%. However, the prevalence of tiers one and two contraception was only 54.2%. There was an unmet need for contraception in 9.5% of participants. The most common method used was male condoms (29%), followed by combined oral contraceptive pills (24.3%).

**Table 3:** Contraceptive prevalence (N = 238)

Variables	Total (N = 238) n (%)
Did not use contraceptives	86 (36.1)
Not sexually active in the past 12 months	70 (81.4)
Sexually active in the past 12 months	16 (18.6)
Use of contraceptive	152 (63.9)
Combined oral contraceptive pills	37 (24.3)
Contraceptive patch	0 (0)
Progesterone-only pills	1 (0.7)
DMPA^a^	9 (5.9)
Contraceptive implant(s)	7 (4.6)
Female Sterilization	31 (20.4)
Male Sterilization	2 (1.3)
Copper IUD^b^	1 (0.7)
LNG-IUS^c^	0 (0)
Male condoms	44 (29)
Female condoms	2 (1.3)
Diaphragm	0 (0)
Withdrawal method	14 (9.2)
Lactation amenorrhea	1 (0.7)
Contraceptive prevalence	
Contraceptive prevalence among sexually active women	90.5 (152/168)
Modern contraceptive prevalence among sexually active women^d^	81.6 (137/168)
Tier 1 and 2 contraceptive prevalence among sexually active women^e^	54.2 (91/168)

^a^Depot medroxyprogesterone acetate

^b^Copper intrauterine device

^c^Levonogestrel intrauterine system

^d^Modern contraceptives including any medical intervention used for contraception [24]

^e^Tier 1 and 2 contraceptive prevalence include sterilization, hormonal contraception, and lactation amenorrhea [25]

### Factors associated with highly effective contraceptive use

DM type was not associated with highly effective contraceptive use (Table 4). Multivariable logistic regression analysis showed that compared to those whose age was ≤ 35 years, those whose age was > 45 years were associated with highly effective contraceptive use, of which the adjusted ORs (95% CI) was 4.88 (1.84, 12.93). Participants who scored higher in the questionnaire about prevention against unplanned pregnancy or regarding confidence in sexual and reproductive planning practice were associated with highly effective contraceptive use, of which the adjusted ORs (95% CIs) were 3.78 (1.88, 7.58) and 3.23 (1.41, 7.38), respectively.

**Table 4:** Factors associated with highly effective contraceptive use by logistic regression among sexually active participants (n = 168)

Factor	Univariable model		Adjusted multivariable model^a^	
	Odds ratio (95% CI)	P-value	Odds ratio (95% CI)	P-value
Type of DM				
Type 1	2.22 (0.66, 7.52)	0.20		
Type 2	Reference			
Age group				
≤ 35 years	Reference		Reference	
> 35–45 years	1.19 (0.57, 2.46)	0.65	1.64 (0.74, 3.63)	0.22
> 45 years	2.57 (1.11, 5.97)	0.03	4.88 (1.84, 12.93)	< 0.01
Age at diabetic diagnosis				
≤ 35 years	Reference			
> 35–45 years	1.68 (0.85, 3.33)	0.14		
> 45 years	3.09 (0.78, 12.29)	0.11		
Address				
Bangkok	1.27 (0.69, 2.32)	0.45		
Others^b^	Reference			
Religions				
Buddhism	0.85 (0.22, 3.29)	0.82		
Others^c^	Reference			
Reimbursement				
Universal Coverage Scheme	1.13 (0.39, 3.28)	0.83		
Social Health Insurance Scheme	0.58 (0.20, 1.69)	0.32		
Self-reimbursement and Missing data	Reference			
King Chulalongkorn Memorial Hospital Officers and Civil Servant Scheme	0.75 (0.28, 2.01)	0.57		
Marital status				
Married	0.81 (0.41, 1.61)	0.55		
Single or divorced	Reference			
Highest education status				
Lower than Bachelor’s degree	Reference			
Bachelor’s degree or higher	0.62 (0.33, 1.15)	0.13		
Occupation and current employment status				
Others^d^	Reference			
Healthcare professional	1.60 (0.68, 3.77)	0.28		
Comorbidities^e^				
Yes	0.58 (0.26, 1.29)	0.18		
No	Reference			
Missing	0.79 (0.39, 1.58)	0.50		
Knowledge score				
< median score	Reference			
≥ median score	0.64 (0.34, 1.21)	0.17		
Attitude toward the negative consequence of sexual activity				
< median score	Reference			
≥ median score	0.95 (0.52, 1.75)	0.88		
Attitude toward benefits of preconception planning				
< Median score	Reference			
≥ Median score	0.72 (0.39, 1.31)	0.28		
Attitude toward barriers to contraception				
< Median score	Reference			
≥ Median score	1.18 (0.63, 2.20)	0.60		
Practice toward a person who affects family planning practices^f^				
< Median score	Reference			
≥ Median score	0.88 (0.48, 1.61)	0.67		
Practice toward prevention against unplanned preganancy^g^				
< Median score	Reference		Reference	
≥ Median score	3.24 (1.72, 6.13)	< 0.01	3.78 (1.88, 7.58)	< 0.01
Practice toward confidence in family planning practice^h^				
< Median score	Reference		Reference	
≥ Median score	2.61 (1.25, 5.44)	0.01	3.23 (1.41, 7.38)	0.01

^a^Adjusted multivariable model including age group, practice toward prevention against unplanned pregnancy, and confidence in family planning practice

^b^Including other provinces in Thailand

^c^Including Christianity, Islam, and no religion

^d^Including students, self-employed, office workers, civil servants, and others

^e^Including diabetic ophthalmopathy, diabetic nephropathy, diabetic neuropathy, ischemic and hemorrhagic stroke, and peripheral arterial disease

^f^Including questions “How does the doctor or nurses’ advice affect your decision to practice family planning?” and “How do others (friends and family, health care providers, mass media) affect your motivation to practice family planning?”

^g^Including questions “When I have sex, I intend to use a birth control method that gives me full protection against unplanned pregnancy” and “When I have sex, I intend to always use some type of birth control to prevent an unplanned pregnancy”

^h^Including question “How confident are you that you are able to practice family planning correctly?

## Discussion

Contraceptive prevalence among sexually active women with DM was as high as 90.5%. This contraceptive prevalence was much higher than reported in the literature [13, 19, 29–31]. However, considering tiers one and two methods, the contraceptive prevalence was only approximately 54.2%. Male condoms and COCs, the most commonly used methods, had a very low perfect-use failure rate [32]. However, both methods may have a high typical usage failure rate [32]. The high prevalence of COCs used in this study was supported by their availability without a prescription. The hesitation among healthcare providers to recommend contraception to women with medical diseases may contribute to using non-prescription methods, such as COCs or condoms [33]. LARC is readily accessible, yet relatively few participants utilized it. Less than half of our participants correctly identified LARC as safe for women with DM; this may explain the low prevalence of LARC use. In addition, about half of the participants had never addressed preconception planning with clinicians. The lack of LARC counseling may be another explanation for our participants’ low LARC utilization [34]. High up-front costs, the stigma of accessing sexual and reproductive planning services among unmarried women, and apprehension of pain connected with LARC adoption may also be contributing factors [35, 36]. Incorporating reproductive health care into DM treatment will increase access to preconception care, which may lead to a greater prevalence of LARC use and a reduction in the rate of unintended pregnancies among women with diabetes [22, 37].

Our participants’ knowledge of pregnancy planning and pregnancy-related risks was insufficient, with a median knowledge score of approximately 40%. Less than half of our participants were aware that insulin is safe during pregnancy, although this observation may be influenced by the large proportion of nulliparous women in the study who may not have received specialized diabetes treatment counseling during pregnancy. This also reflects a broader issue of inadequate knowledge of glycemic management during pregnancy within the cohort. Less than half of the participants realized that IUDs were safe for women with DM. Despite evidence to the contrary, misconceptions regarding IUD safety corroborate our findings and the rarity of IUD usage among Thais [38, 39]. Women who were diagnosed with DM at a younger age, not being Buddhist, were married, had higher educational levels, and worked as medical personnel were associated with higher knowledge scores, focusing on pregnancy planning and pregnancy-related risks. The higher knowledge scores among women with an earlier age at DM diagnosis may be due to more frequent exposure to the healthcare system over a longer DM history. While we observed that non-Buddhist participants scored higher, this finding warrants further investigation to elucidate the underlying factors. Religion is a complex variable that may serve as a proxy for various other factors, such as socioeconomic status and educational attainment, which in turn could influence the participants' knowledge levels. Married women may seek more information about pregnancy, realizing they are at risk of pregnancy. Our findings are consistent with a French multicenter cross-sectional study that found that women with higher educational levels had more knowledge about pregnancy planning and pregnancy-related risks [29]. Highly educated individuals and medical personnel are more likely to seek health-related information and can distinguish or judge the reliability of information sources [40].

Detailed perinatal care is required for women with preexisting diabetes; this care includes frequent blood glucose monitoring, insulin injections, and evaluation of microvascular complications. Preconception planning, which contributes to minimizing adverse pregnancy outcomes, will aid these vulnerable women in preparing for these time-consuming procedures [10]. However, only half of our participants reported receiving preconception care, and only half of the individuals chose contraception based on recommendations from their healthcare professionals. Multispecialty care fragmentation impedes access to reproductive healthcare for participants in tertiary care settings [41]. A substantial gap exists in integrated care between specialties, especially in the multisystemic nature of DM [42].

Our results showed that women aged > 45 years and those with higher practice scores toward the prevention of unplanned pregnancy or practice scores toward confidence in sexual and reproductive planning practice were associated with higher odds of using highly effective contraception. The most prevalent highly effective contraceptive use in our setting was sterilization, which is always associated with women’s age [43, 44]. A previous study showed that increasing age, higher income levels, and receiving contraceptive counseling are related to contraceptive use among women with DM [30]. Incorporating reproductive healthcare into the management of diabetes will improve the quality of care, reduce healthcare expenditures, and boost patient satisfaction [4].

In our context, internists/endocrinologists and obstetricians/gynecologists should collaborate to provide annual preconception care, highlighting the safety of LARC use among all reproductive-aged diabetic women who do not wish to become pregnant. Consideration and implementation of effective multimedia technologies, such as educational interventions to increase awareness of glycemic control and appropriate preconception information among women with DM, have been described as beneficial in prior publications [21, 44]. As of the fiscal year 2022, the Ministry of Public Health and the National Security Office of Thailand expanded the LARC reimbursement policy to cover all Thai women of reproductive age. Women with DM could have improved access to reproductive healthcare services if these obstacles were removed.

To the best of our knowledge, very few publications have focused on reproductive health care among women with DM in Thailand [17]. The strengths of this study are as follows. Women with type 1 and 2 DM were included in this study. We investigated the differences in knowledge regarding preconception care focusing on pregnancy planning, pregnancy-related risks, and factors associated with highly effective contraceptive use because these two conditions may be associated with different pregnancy outcomes [46]. However, because this study comprised a limited number of women with type 1 DM, the power of the test has been compromised. This could limit our ability to draw meaningful conclusions or detect significant differences (if any). Diabetes management and participant comorbidities were verified to confirm the correctness. This study was conducted at a tertiary referral center and residency training site in various clinical areas, including complicated DM cases. Finally, almost all contraceptives were readily available.

This study has several limitations; this was a descriptive cross-sectional study conducted at a single center. Therefore, generalizability is restricted to urban and tertiary settings. While the primary focus of this study was to assess the impact of diabetes mellitus on preconception care knowledge, attitude, and practice, it is noteworthy that 63.5% of our participants had other comorbidities, which could also influence their preconception care knowledge, attitude, and practice. Due to the coronavirus disease of 2019 situation in Thailand, the eligibility of participants was determined via short telephone interviews. Since only phone-reachable women were included in this study, selection bias must be addressed. Due to the nature of the survey, it was only possible to collect limited data. An in-depth interview may help us better understand the participants' choice of contraception and their reasons for not using it when they are at risk of pregnancy. In addition, few individuals used LARC in conjunction with highly effective contraception, restricting us from performing a subgroup analysis that concentrated on variables related to LARC usage. Since we hypothesized that this is a high-risk group for unintended pregnancy, we focused primarily on individuals who did not desire to conceive. Finally, we developed the questionnaire based on existing research that did not specify the score cutoff for each component [13, 19, 29–31].

## Conclusions

The contraceptive prevalence among women with DM was high; however, only half of our participants used tier one or two contraceptives. Women with DM have some unmet demands for contraception. Furthermore, knowledge regarding preconception planning was limited, and only half of the participants discussed it with their physicians. In our setting, there was a significant gap in coordinated care among specialties; incorporating reproductive health care into DM treatment will increase access to preconception care.

### Supplementary Information

**Additional file 1: Table S1.** Percentage of corrected response to pregnancy planning and pregnancy-related risks (N = 238). **Table S2.** Percentage of correct response to pregnancy planning and pregnancy-related risks (N = 238). **Table S3.** Attitude toward preconception care among women with diabetes (N = 238). **Table S4.** Practice toward contraceptive use among women with diabetes (N = 238)

## Data Availability

The datasets that were generated or analyzed during the current study are available from KP upon reasonable request.
