# Supplementary material for: Reproductive care in Thai women with diabetes mellitus: a descriptive cross-sectional study
Source: Reprod Health. 2023 Oct 12;20:152. doi: 10.1186/s12978-023-01694-w (PMC10571380; doi:10.1186/s12978-023-01694-w)
Supplement: Supplementary file 1 — Additional file 1: Table S1. Percentage of corrected response to pregnancy planning and pregnancy-related risks (N = 238). Table S2. Percentage of correct response to pregnancy planning and pregnancy-related risks (N = 238). Table S3. Attitude toward preconception care among women with diabetes (N = 238). Table S4. Practice toward contraceptive use among women with diabetes (N = 238) [file 12978_2023_1694_MOESM1_ESM.docx]

**Additional file 1: Table S1 Disease status and associated comorbidities among diabetic women (N = 238)**

| **Variables** | **Total**  **N = 238 n (%)** |
| --- | --- |

| **Age at diabetes diagnosis** |  |
| --- | --- |
| - Mean±SD | 28.9±13.4 |
| - Median (IQR) | 32 (20) |

| **Age group at diabetes diagnosis** |  |
| --- | --- |
| =< 19 years | 56 (23.5) |
| > 19 – 24 years | 18 (7.6) |
| > 24 – 35 years | 73 (30.7) |
| > 35 – 45 years | 77 (32.4) |
| > 45 years | 14 (5.9) |
| **HbA1C** |  |
| Recent |  |
| - Mean±SD | 7.2**±**1.6 |
| - Median (IQR) | 6.9 (1.7) |
| Past 3-6 months |  |
| - Mean±SD | 7.4**±**1.8 |
| - Median (IQR) | 6.9 (1.4) |
| Average |  |
| - Mean±SD | 7.3**±**1.6 |
| - Median (IQR) | 7.0 (1.6) |
| **Complications of disease** |  |
| Without complication | 108 (45.4) |
| With complications | 48 (20.2) |
| Missing data | 82 (34.5) |
| **Ischemic stroke** |  |
| No | 147 (61.8) |
| Yes | 9 (3.8) |
| Missing data | 82 (34.5) |
| **Hemorrhagic stroke** |  |
| No | 152 (63.9) |
| Yes | 4 (1.7) |
| Missing data | 82 (34.5) |
| **Coronary heart disease** |  |
| No | 138 (58) |
| Yes | 18 (7.6) |
| Missing data | 82 (34.5) |
| **Peripheral arterial disease** |  |
| No | 132 (55.5) |
| Yes | 24 (10.1) |
| Missing data | 82 (34.5) |
| **Diabetic ophthalmopathy** |  |
| No | 118 (49.6) |
| Yes | 38 (16) |
| Missing data | 82 (34.5) |
| **Diabetic nephropathy** |  |
| No | 119 (50) |
| Yes | 37 (15.6) |
| Missing data | 82 (34.5) |
| **Diabetic neuropathy** |  |
| No | 127 (53.4) |
| Yes | 29 (12.2) |
| Missing data | 82 (34.5) |
| **Current diabetic medication** | 226 (95) |
| **Oral medications** |  |
| No | 46 (19.3) |
| Yes | 190 (79.8) |
| Missing data | 2 (0.8) |
| **Injection** |  |
| No | 137 (57.6) |
| Yes | 100 (42) |
| Missing data | 1 (0.4) |
| **Combination^a^** |  |
| No | 175 (73.5) |
| Yes | 63 (26.5) |
| **Other comorbidities** |  |
| No | 67 (28.2) |
| Yes | 151 (63.5) |
| Missing data | 20 (8.4) |
| **Hypertension** |  |
| No | 123 (51.7) |
| Yes | 95 (39.9) |
| Missing data | 20 (8.4) |
| **Dyslipidemia** |  |
| No | 125 (52.5) |
| Yes | 93 (39.1) |
| Missing data | 20 (8.4) |
| **Hematologic disease^b^** |  |
| No | 214 (89.9) |
| Yes | 4 (1.7) |
| Missing data | 20 (8.4) |
| **Endocrinologic disease^c^** |  |
| No | 205 (86.1) |
| Yes | 13 (5.5) |
| Missing data | 20 (8.4) |
| **Psychiatric disease^d^** |  |
| No | 209 (87.8) |
| Yes | 9 (3.8) |
| Missing data | 20 (8.4) |
| **Neurologic disease**^e^ |  |
| No | 214 (89.9) |
| Yes | 4 (1.7) |
| Missing data | 20 (8.4) |
| **Rheumatologic disease^f^** |  |
| No | 217 (91.2) |
| Yes | 1 (0.4) |
| Missing data | 20 (8.4) |
| **Cancer^g^** |  |
| No | 211 (88.7) |
| Yes | 7 (2.9) |
| Missing data | 20 (8.4) |
| **Cardiac disease** |  |
| No | 211 (88.7) |
| Yes | 7 (2.9) |
| Missing data | 20 (8.4) |
| **Other diseases^h^** |  |
| No | 174 (73.1) |
| Yes | 44 (18.5) |
| Missing data | 20 (8.4) |

^~~a~~^Including both oral medication and injection

^b^Including anemia, autoimmune hemolytic anemia, polycythemia, and thrombocytopenia

^c^Including hyperthyroid, hypothyroid, thyroid nodule, and autoimmune thyroiditis

^d^Including depression, bipolar, anxiety disorder, panic disorder, and schizophrenia

^e^Including stroke, epilepsy, myasthenia gravis, multiple sclerosis, and migraine

^f^Including systemic lupus erythematosus and rheumatoid arthritis

^g^Including thyroid cancer, breast cancer, colonic cancer, and lymphoma

^h^Including fatty liver, obstructive sleep apnea, gastroesophageal reflux

**Additional file 1: Table S2 Percentage of correct response to** **pregnancy planning and pregnancy-related risks**

**(N = 238)**

| **Diabetes and pregnancy knowledge** | **Total (N = 238)** | |
| --- | --- | --- |
|  | **Correct n (%)** | **Incorrect/ I don’t know**  **n (%)** |
| 1. All insulins are suitable for use during pregnancy  (True = correct) | 108 (45.4) | 130 (54.6) |
| 2. Women with diabetes should get advice from a healthcare professional  (True = correct) | 213 (89.5) | 25 (10.5) |
| 3. Women with diabetes should take the same amount of folic acid as all other women planning a pregnancy  (True = correct) | 51 (21.4) | 187 (78.6) |
| 4. Before becoming pregnant, diabetic women should be screened for disease complications and re-evaluated every trimester |  |  |
| Diabetic retinopathy (True = correct) | 154 (64.7) | 84 (35.3) |
| Diabetic nephropathy (True = correct) | 157 (65.97) | 81 (34.03) |
| 5. Before becoming pregnant, your blood glucose level should be between… |  |  |
| 1 hour before meals |  |  |
| Know | 79 (33.2) |  |
| Do not know | 159 (66.8) |  |
| If you know, how much (95 = correct) | 18 (7.56) | 220 (92.4) |
| 1 hour postprandial |  |  |
| Know | 67 (28.2) |  |
| Do not know | 171 (71.8) |  |
| If you know, how much (120 = correct) | 11 (4.6) | 227 (95.4) |
| 6. Before becoming pregnant, ideally your HbA1c should be below |  |  |
| Know | 34 (14.3) |  |
| Do not know | 204 (85.7) |  |
| If you know, how much (6.5 = correct) | 8 (3.4) | 230 (96.6) |
| 7. If you have type 2 diabetes and are planning to become pregnant, you may need to change from insulin tablets to injections (True = correct) | 76 (31.9) | 162 (68.1) |
| 8. High blood glucose levels during pregnancy do not increase the risk of problems for |  |  |
| The mother (False = correct) | 137 (54.6) | 101 (42.4) |
| The babies (False = correct) | 149 (62.6) | 89 (37.4) |
| 9. Women with diabetes have an increased risk of having |  |  |
| A large baby (True = correct) | 132 (55.5) | 106 (44.5) |
| Making delivery more difficult (True = correct) | 119 (50) | 119 (50) |
| Baby with birth defects (True = correct) | 146 (61.3) | 92 (38.7) |
| Risk of miscarriage (True = correct) | 135 (56.7) | 103 (43.3) |
| Perception care score of pregnancy planning and pregnancy-related risks |  |  |
| - Mean±SD | 6.8±3.5 |  |
| - Median (IQR) | 7 (6) |  |

**Additional file 1: Table S3 Attitude toward** **preconception care among diabetic women (N = 238)**

| **Factor** | **Total**  **N = 238 n (%)** |
| --- | --- |
| **Susceptible to negative outcomes of sexual activity** (Score: 3-15) |  |
| - Mean±SD | 12.5±2.7 |
| - Median (IQR) | 13 (4) |
| **Severity to negative maternal outcomes of sexual activity** (Score: 4-20) |  |
| - Mean±SD | 17.0±3.1 |
| - Median (IQR) | 17 (4) |
| **Severity to negative perinatal outcomes of sexual activity** (Score: 5-25) |  |
| - Mean±SD | 20.8±3.9 |
| - Median (IQR) | 20.5 (6) |
| **Benefit of preconception planning** (Score: 4-20) |  |
| - Mean±SD | 17.3±3.1 |
| - Median (IQR) | 18 (4) |
| **Barriers to contraception** (Score: 5-25) |  |
| - Mean±SD | 13.2±4.0 |
| - Median (IQR) | 13 (4) |

**Additional file 1: Table S4 Practice toward contraceptive use among diabetic women (N = 238)**

| **Factor** | **Total**  **N = 238 n (%)** |
| --- | --- |
| **Cues to action** (Score: 4-20) |  |
| - Mean±SD | 13.1±3.6 |
| - Median (IQR) | 14 (5) |
| **Intention to contraceptive use for protection against unplanned pregnancy** (Score: 2-10) |  |
| - Mean±SD | 4.6±2.9 |
| - Median (IQR) | 5 (6) |
| **Confidence in current contraceptive use** (Score: 1-5) |  |
| - Mean±SD | 2.2±1.3 |
| - Median (IQR) | 2 (2) |
| **Have you ever discussed pregnancy planning during visits with your internist or endocrinologist?** |  |
| Yes | 128 (53.8) |
| No | 110 (46.2) |
| **Who recommended the type of contraceptive that you use?** |  |
| Internist/ endocrinologist | 69 (29.0) |
| Obstetricians and gynecologists | 54 (22.7) |
| Partner | 78 (32.8) |
| Family member | 27 (11.3) |
| Friend | 14 (5.9) |
| Yourself | 123 (51.7) |
| Others^a^ | 21 (8.82) |
| **What makes you more confident in practicing family planning?** |  |
| Partner | 126 (52.94) |
| Family member | 44 (18.49) |
| Friend | 26 (10.92) |
| Yourself | 118 (49.58) |
| Others^a^ | 47 (19.75) |

^a^Including mass media and the internet
